# Genome-Wide Landscapes of Human Local Adaptation in Asia

**DOI:** 10.1371/journal.pone.0054224

**Published:** 2013-01-22

**Authors:** Wei Qian, Lian Deng, Dongsheng Lu, Shuhua Xu

**Affiliations:** Max Planck Independent Research Group on Population Genomics, Chinese Academy of Sciences and Max Planck Society (CAS-MPG) Partner Institute for Computational Biology, Shanghai Institutes for Biological Sciences, Chinese Academy of Sciences, Shanghai, China; University of Cambridge, United Kingdom

## Abstract

Genetic studies of human local adaptation have been facilitated greatly by recent advances in high-throughput genotyping and sequencing technologies. However, few studies have investigated local adaptation in Asian populations on a genome-wide scale and with a high geographic resolution. In this study, taking advantage of the dense population coverage in Southeast Asia, which is the part of the world least studied in term of natural selection, we depicted genome-wide landscapes of local adaptations in 63 Asian populations representing the majority of linguistic and ethnic groups in Asia. Using genome-wide data analysis, we discovered many genes showing signs of local adaptation or natural selection. Notable examples, such as *FOXQ1*, *MAST2*, and *CDH4*, were found to play a role in hair follicle development and human cancer, signal transduction, and tumor repression, respectively. These showed strong indications of natural selection in Philippine Negritos, a group of aboriginal hunter-gatherers living in the Philippines. *MTTP*, which has associations with metabolic syndrome, body mass index, and insulin regulation, showed a strong signature of selection in Southeast Asians, including Indonesians. Functional annotation analysis revealed that genes and genetic variants underlying natural selections were generally enriched in the functional category of alternative splicing. Specifically, many genes showing significant difference with respect to allele frequency between northern and southern Asian populations were found to be associated with human height and growth and various immune pathways. In summary, this study contributes to the overall understanding of human local adaptation in Asia and has identified both known and novel signatures of natural selection in the human genome.

## Introduction

High-throughput DNA genotyping and sequencing technologies and novel statistical approaches advanced genome-wide detection of molecular signature of natural selection in the human genome [1]–[9]. In previous studies, a large number of loci have been identified as candidates for local adaptation in human populations on a broad continental scale, providing a good indication of gene-environment interactions in human evolution [10]–[13]. Remarkable examples showing strong signs of positive selection include *HBB* and *G6PD*, whose mutants and deficiency alleles were found to confer resistance to malaria respectively, *LCT*, whose variant allows lactose tolerance to persist throughout adulthood, *SLC24A5*, which contributes to skin pigmentation diversity, and *EDAR*, one of whose polymorphisms may cause variation in hair morphology [6], [14]–[21].

However, most of the previous genome-wide studies of natural selection have concentrated on population samples collected from international collaborative efforts such as the Human Genome Diversity Panel (HGDP), the International HapMap Project, and the 1000 Genome Project [22]–[24]. Up until now, samples from five major population groups (populations located in or with ancestry from Europe, East Asia, South Asia, West Africa, and the Americas) have been included in those large projects and databases. Recently, a few genome-wide investigations of the role of natural selection have been performed in Asian populations. For instance, there have been studies of diversity of the *NAT2* gene supporting acetylation in human adaptation to farming in Central Asia, positive selection on *NRG-ERBB4* pathway in Middle East, natural selection on an *ABCC11* SNP determining earwax type in East Asians, and *EPAS1* and *EGLN1*, which are likely to be responsible for high-altitude adaptations in Tibetans [25]–[28]. Southeast Asia is home to a great deal of humanity's genetic diversity. Although this vast area has been crucial to human history [29]–[33], it has been greatly underrepresented in similar efforts worldwide [6], [12], [13], [26], [34]–[37].

We therefore attempted to provide the first comprehensive landscape of local adaptations in Southeast Asia using genome-wide analysis of 63 populations obtained from the HUGO Pan-Asian SNP consortium, where 54,794 autosomal single-nucleotide polymorphisms (SNPs) in 1513 individuals were genotyped using Affymetrix GeneChip Human Mapping 50K Xba Array [38], [39].

Taking advantages of the dense population samples, we drew a general yet comprehensive picture of human local adaptation, particularly in Southeast Asia. First, we characterized an overview of putative local adaptation signals using between-population comparisons, which provided a guide for further, finer-scale examinations. However, since the marker density was not sufficiently high in our data, we mainly focused on allele-frequency-based analysis rather than haplotype-based analysis. We classified populations into several sub-groups according to the genetic structures revealed in a previous study [39]. To identify putative candidate genes underlying possible selection, we first employed a sliding window-based strategy and set a 1% genome-wide cutoff of population differentiation in allele frequency as indication of natural selection. As a result, we selected the top 200 genes containing the strongest signals as indicated by SNPs within windows in each pair (see Methods). Second, we performed functional enrichment analysis to provide biological interpretation of those candidates of natural selection identified in this study. Finally, a list of top candidate genes was obtained by gene ranking analysis and we highlighted a couple of the strongest signals, which coherently carried a significant number of group-specific variants. These are more likely to be associated with local adaptation, and should merit further investigation in future studies.

## Results

### Overview of Signals

After removing some populations showing evident admixture as reported in a previous study [39], we obtained a dataset composed of 54,794 SNPs genotyped on 1513 individuals, representing 63 Asian populations [38]. For the sake of more powerful scans for local adaptation signs, we decided to merge certain populations in order to increase the sample size. According to their genetic structures revealed by a previous study, we classified those individuals into nine sub-groups, including, from north to south, Japanese&Korean, Han Chinese, SouthernChinese&Thai1, SouthernChinese&Thai2, Indonesian, Philippine Negrito, Southeast Asian, Malaysian Negrito, and Indian (Table S1) [39]. Each group contained individuals who were closely related genetically, and individuals in different groups inhabited various environments. These in turn shaped their genomes and left signs of local adaptation.

In order to detect signatures of local adaptation on a genome-wide scale, we used an allele-frequency-based approach involving pairwise comparison of those nine groups. First, we calculated population differentiation, F_ST_, of genome-wide SNP markers in each pair. As the total sample size of each group was not always similar, some groups might have much larger or smaller sample size than others. We randomly sampled the same number of individuals from each group so that the sample size of groups under comparison is comparable. The most commonly used means of identifying putative signals of selection has been the outlier-based method [3], [5], which requires genome-wide data to distinguish signatures of natural selection from demographic history. Further, selection signals in the context of gene level were detected by rule of containing significantly high F_ST_ values within genes. In order to control the bias resulting from the number of genotyped SNPs or the size of genes, here we employed a sliding window strategy (see Methods) and finally picked out top 200 genes in each of the 36 comparisons, which were considered as putative candidates of local adaptation. To delineate the overall picture of putative signals of local adaptation in the 9 groups, we calculated global F_ST_ of total SNP markers and gave maximum and minimum F_ST_ values of the candidate genes selected as putative signals in each group-pair comparison (Table 1). As expected, the result of global population differentiation from Table 1 showed that Negritos from both the Philippines and Malaysia had much greater genetic differences than other Asian populations. Indian populations showed the most remarkable global differentiation among the Asian populations; their genomes harbored a considerable amount of Caucasian ancestry, which is considerably different from Asian ancestry.

**Table 1:** Summary of signatures of population differentiation measured by F_ST_ among 9 groups.

Group Name	JK	Han	SCT1	SCT2	INDO	PN	SEA	MN	IND
**JK**	-	**0.0048** [0.0047,0.0049]	**0.0156** [0.0153,0.0159]	**0.0138** [0.0136,0.0140]	**0.0394** [0.0388,0.0400]	**0.038** [0.0374,0.0386]	**0.0178** [0.0176,0.0181]	**0.062** [0.0611,0.0628]	**0.0706** [0.0698,0.0715]
**Han**	**0.14/0.06**	-	**0.0064** [0.0062,0.0066]	**0.0051** [0.0050,0.0052]	**0.0341** [0.0336,0.0346]	**0.0323** [0.0317,0.0329]	**0.0094** [0.0092,0.0095]	**0.0567** [0.0558,0.0575]	**0.0707** [0.0697,0.0716]
**SCT1**	**0.37/0.16**	**0.28/0.11**	-	**0.0088** [0.0086,0.0091]	**0.0378** [0.0372,0.0384]	**0.0371** [0.0365,0.0378]	**0.0137** [0.0134,0.0140]	**0.059** [0.0581,0.0599]	**0.0733** [0.0723,0.0743]
**SCT2**	**0.28/0.11**	**0.22/0.06**	**0.26/0.13**	-	**0.0298** [0.0294,0.0302]	**0.0303** [0.0297,0.0308]	**0.0079** [0.0077,0.0080]	**0.0449** [0.0443,0.0456]	**0.066** [0.0651,0.0668]
**INDO**	**0.51/0.27**	**0.50/0.24**	**0.58/0.28**	**0.46/0.22**	-	**0.0257** [0.0252,0.0261]	**0.0207** [0.0203,0.0210]	**0.0539** [0.0532,0.0548]	**0.0575** [0.0568,0.0583]
**PN**	**0.98/0.32**	**0.98/0.30**	**0.98/0.33**	**0.98/0.28**	**0.98/0.27**	-	**0.0195** [0.0191,0.0199]	**0.0585** [0.0576,0.0594]	**0.0591** [0.0582,0.0599]
**SEA**	**0.45/0.13**	**0.36/0.09**	**0.46/0.16**	**0.38/0.07**	**0.32/0.17**	**0.92/0.23**	-	**0.0458** [0.0452,0.0465]	**0.06** [0.0592,0.0608]
**MN**	**0.55/0.35**	**0.53/0.33**	**0.58/0.35**	**0.49/0.28**	**0.53/0.32**	**0.98/0.38**	**0.48/0.29**	-	**0.0709** [0.0700,0.0719]
**IND**	**0.74/0.41**	**0.73/0.39**	**0.74/0.43**	**0.63/0.37**	**0.60/0.34**	**0.90/0.38**	**0.71/0.34**	**0.69/0.40**	-

Note: Groups are abbreviated as: JK – Japanese&Korean, Han – Han Chinese, SCT1 – SouthernChinese&Thai1, SCT2 – SouthernChinese&Thai2, INDO – Indonesian, PN – PhilippineNegrito, SEA – SoutheastAsian, MN – MalaysianNegrito, IND – Indian. Slash represents unknown gene function. The upper matrix data represents the global F_ST_ of each comparing group pair. Data in the square bracket represents 90% confidence intervals obtained by bootstrapping. The lower matrix data shows the maximum F_ST_/minimum F_ST_ among 200 selected candidate genes for signals of population differentiation in each group pair.

To confirm the selection of genomic regions indicated by the F_ST_ approach, we used another allele-frequency-based method called cross-population composite likelihood ratio test (XP-CLR) [40]. By integrating information of neighboring markers, this method is believed to be more powerful in detecting signals of natural selection. Detailed results of XP-CLR analysis are available online in supporting materials (Text S1, Table S2). We found that many candidate genes identified by F_ST_-based approach also appeared on the list produced by the XP-CLR test (Table S3). For example, *FOXQ1* and *PIK3R3* showed significant signatures all along in the comparisons between Philippine Negritos and any other Asian populations; *AMZ1* appeared frequently in the comparisons between SouthernChinese&Thai1 and other Asian groups such as Negritos from Malaysia, Southeast Asians and Han Chinese. More details will be discussed in the following sections.

### Functional analysis and an overview

**Figure 1:**
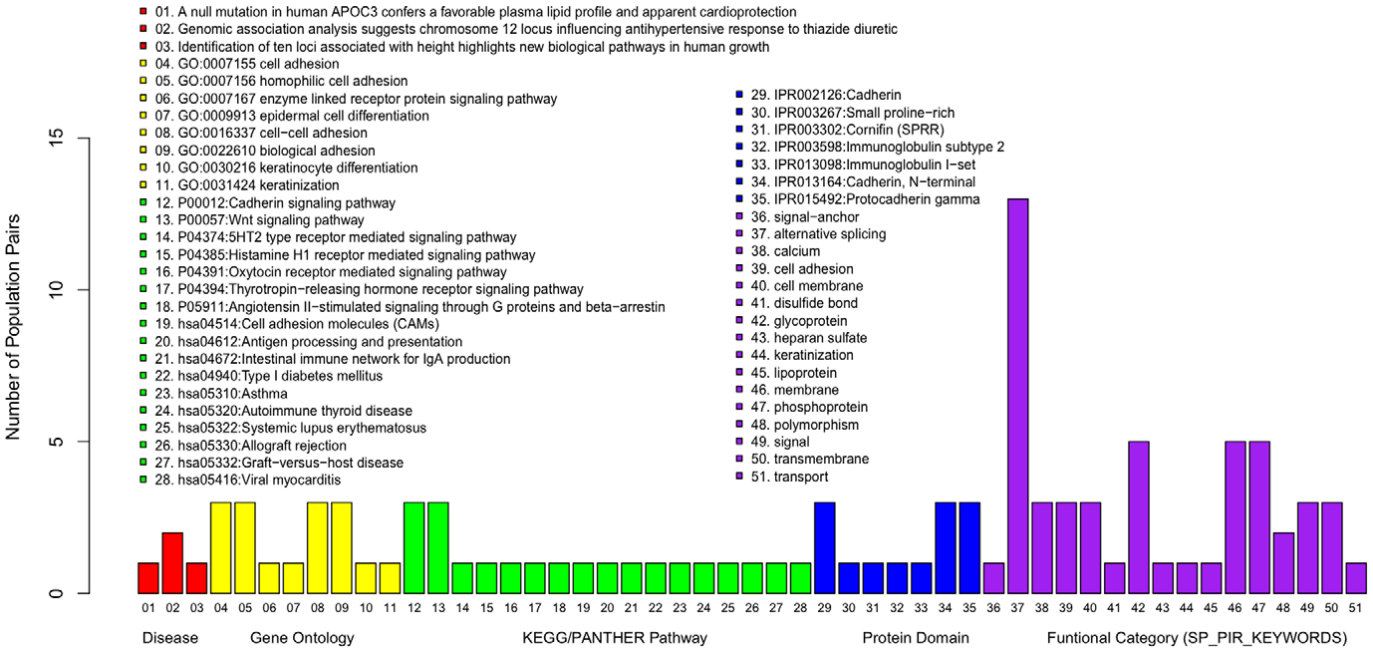
Functional categories among pairs of different population groups. Functional analysis was performed using DAVID annotation. The distribution of significant functional categories among the comparisons covered all the terms meeting a criterion of Benjamini FDR corrected p<0.05. Each color represents one functional term. The y-axis represents the frequency at which the functional term showing significant enrichment occurred in the comparisons of the nine groups in pairs.

In order to biologically interpret and characterize the local adaptation signatures in Asian populations, which were mainly inferred from statistical analysis, we further conducted a functional enrichment analysis of the 200 candidate genes whose SNP markers exhibited extremely significant between-population allele frequency differentiation against the whole genome. Functional annotations (see Methods) revealed that the functional category of alternative splicing was frequently presented in most pairs (Figure 1, Benjamini FDR corrected p<0.05), suggesting its vital and general role in the local adaptations of most Asian populations. This category was most significantly enriched in the group pairs involving northern populations in East Asia (e.g. Japanese&Korean, and Han Chinese), southern populations in South Asia (Indian) and Southeast Asian populations (East Indonesian and Negrito groups from the Philippines and Malaysia) (Table S4).

On the other hand, we observed considerable group specific or regional selection signatures in our data. For example, specific to Indonesian populations, a series of candidate genes displayed significant enrichment in cell adhesion-related terms (Table S4, Benjamini FDR corrected p<0.001). Signals of selection came from a superfamily of Cadherins, in which glycoproteins involved in Ca^2+^-mediated cell-cell adhesion and their evolving domain structures serve to specific cell adhesion and intricate cell signaling [41]. These findings were also supported by several other significantly enriched functional keywords (Table S4) such as those of signal, calcium and glycoprotein (Benjamini FDR corrected p<0.001). These cadherin-related terms (Figure 1) most frequently showed enrichment in the comparisons between southern groups (Indonesians) and northern groups (Japanese&Korean, Han Chinese, SouthernChinese&Thai2). In addition, comparisons of northern populations (Han Chinese, Japanese&Korean) and the other southern populations (SouthernChinese&Thai1, SouthernChinese&Thai2, Southeast Asian) revealed that immunoglobulin (Ig) subtype 2 domain and plenty of immune-associated pathways were significantly differentiated between north and south, such as pathways of graft-versus-host disease, autoimmune thyroid disease, asthma and viral myocarditis, and pathways of allograft rejection, antigen processing and presentation, and intestinal immune network for IgA production (Benjamini FDR corrected p<0.05). These results suggested that the significant difference between northern populations and southeast populations in Asia possibly resulted from the local adaptation of immune system, which might have been subjected to considerable natural selection.

The divergence of Philippine Negritos from most of the other Asian populations (Han Chinese, Japanese&Korean, Indian, Southeast Asian) was best illustrated by our functional analysis (Table S4). The OMIM disease analysis revealed that genes, with a great number of genetic variants showing large allele frequency difference between Japanese&Korean populations and Negritos from the Philippines, were members of ten known loci associated with biological pathways of human height and growth (Benjamini FDR corrected p = 0.03), indicating a local adaptation of specific stature of Negritos residing in the Philippines. Another evidently enriched disease trait of cardioprotection was observed in the comparison between Philippine Negritos and Han Chinese, involving genes with a null mutation favorable for plasma lipid profile (Benjamini FDR corrected p = 0.04). And functional category of keywords suggested that signals of natural selection were enriched for phosphoproteins, which were identified in Philippine Negritos compared with Indians and Southeast Asian populations (Benjamini FDR corrected p<0.05).

Another group of Negritos in Malaysia showed a great variation in keratinocyte differentiation and epidermal cell differentiation compared with Indonesian population (Benjamini FDR corrected p<0.01). There were a series of KEGG signaling pathways showing enrichment of divergence between the Negritos and Indians (Table S4), for example, pathways of histamine H1 receptor mediated signaling and angiotensin II-stimulated signaling through G proteins and beta-arrestin (Benjamini FDR corrected p<0.001). These functional variations were best represented by *PLCG2* with the SNP in the gene reaching F_ST_ of 0.55. These findings indicated that signaling pathways have been under natural selection and might have played an important role in Negritos adapting to local environment in Malaysia.

In summary, giving clues to biological basis of regional natural selection, our functional analyses provided a great deal of insight into human local adaptation based on allele frequency differentiation between populations. A similar procedure of functional analysis was also performed upon signals identified by XP-CLR analysis (Text S1).

### Identification of candidate genes underlying local adaptation

#### Screening of the top candidates

In order to better understand selective pressures upon Asian populations that might not have previously been evaluated, we ranked candidate genes according to the highest F_ST_ value among that of all the SNP markers within it. We chose the top ten candidates from each group pair, as shown in Table S5, and assumed that these candidates were more likely to have been subjected to regional natural selection.

Considering the many group comparisons and candidate genes, we only highlighted the top candidate gene from each pair, as shown in Table 2. Screening the list of those outstanding selection candidates, we found that most of them showed significant enrichment in our functional analysis (Table S4) such as alternative splicing, and were found to have an association with human morphology (Table 2). For example, most of the top candidate genes in the comparison of the populations from relatively northern regions (SouthernChinese&Thai2, Han Chinese, Japanese&Korean) and southern regions of Asia (Indian, Malaysian Negrito, and Southeast Asian) belong to the category of alternative splicing, including *MLKL*, *PPP1CC*, *DAPP1*, and *ABCA12*.

**Table 2:** Top 1 candidate genes identified among 36 pairs of Asian populations.

Group Pair	Gene	Chromosome	No.SNPs	Top SNP	Alleles	Max F_ST_	GO Function
SCT1_SCT2	GALNTL6	4	10	rs6811723	T/C	0.265	O-Glycan biosynthesis, carbohydrate metabolism, protein metabolism and modification, smoking cessation.
SCT1_IND	TTLL11	9	1	rs10513414	T/C	0.738	Ligase activity, cell projection.
SCT1_INDO	AMZ1	7	3	rs798527	T/C	0.578	Proteolysis.
SCT1_MN	AMZ1	7	3	rs798525	A/C	0.584	Proteolysis.
SCT1_PN	FOXQ1	6	1	rs951318	A/G	0.976	Hair follicle development/morphogenesis, epidermis and ectoderm development.
SCT1_SEA	DAPP1	4	1	rs10516445	C/G	0.464	Phosphorus metabolic process.
SCT2_IND	MLKL	16	4	rs4888473	G/A	0.633	Protein phosphorylation and modification.
SCT2_INDO	MTTP	4	3	rs2851292	G/T	0.461	Lipid and protein transport, serum cholesterol level.
SCT2_MN	PPP1CC	12	1	rs11066232	C/T	0.492	Cell division, metabolic processes of glycogen, phosphorus and hexose.
SCT2_PN	FOXQ1	6	1	rs951318	A/G	0.977	Hair follicle development/morphogenesis, epidermis and ectoderm development.
SCT2_SEA	MTTP	4	3	rs2851292	G/T	0.383	Lipid and protein transport, serum cholesterol level.
Han_SCT1	C1orf140	1	1	rs851195	T/C	0.281	/
Han_SCT2	VEGFC	4	6	rs921879	C/G	0.223	Angiogenesis, blood vessel development, regulation of amino acid phosphorylation and neuroblast proliferation, cell motion, cell surface receptor linked signal transduction, embryonic morphogenesis, epithelium development.
Han_IND	ABCA12	2	4	rs1250233	G/A	0.728	Lipid transport and localization, cellular homeostasis,.
Han_INDO	FMO6P	1	4	rs7527462	C/G	0.496	/
Han_MN	BC035398	10	3	rs1998643	A/G	0.532	/
Han_PN	FOXQ1	6	1	rs951318	A/G	0.977	Hair follicle development/morphogenesis, epidermis and ectoderm development.
Han_SEA	DAPP1	4	1	rs10516445	C/G	0.359	Phosphorus metabolic process.
INDO_IND	TTLL11	9	2	rs10513414	T/C	0.602	Ligase activity, cell projection.
INDO_MN	AK096329	1	6	rs477289	A/C	0.521	/
INDO_PN	FOXQ1	6	2	rs951318	A/G	0.977	Hair follicle development/morphogenesis, epidermis and ectoderm development.
INDO_SEA	YTHDC2	5	2	rs182988	T/A	0.323	ATP binding, DNA/RNA helicase.
JK_SCT1	A2BP1	16	40	rs10500343	C/T	0.366	RNA processing and localization.
JK_SCT2	GRIP1	12	7	rs7132697	T/A	0.276	Protein localization, regulation of gene expression, steroid hormone receptor signaling pathway, androgen receptor signaling pathway, regulation of nitrogen compound metabolic process, regulation of RNA metabolic process.
JK_Han	DIAPH3	13	8	rs300310	T/C	0.147	Cytoskeleton organization.
JK_IND	ABCA12	2	4	rs1250233	G/A	0.743	Lipid transport and localization, cellular homeostasis
JK_INDO	MTTP	4	3	rs10516441	G/A	0.509	Lipid and protein transport, serum cholesterol level.
JK_PN	FOXQ1	6	1	rs951318	A/G	0.977	Hair follicle development/morphogenesis, epidermis and ectoderm development.
JK_SEA	MTTP	4	3	rs10516441	G/A	0.449	Lipid and protein transport, serum cholesterol level.
JK_MN	AX747662	1	7	rs10489392	A/G	0.484	/
MN_IND	CCDC104	2	3	rs782603	A/G	0.69	Membrane-enclosed lumen.
PN_IND	FOXQ1	6	2	rs951318	A/G	0.897	Hair follicle development/morphogenesis, epidermis and ectoderm development.
PN_MN	FOXQ1	6	1	rs951318	A/G	0.979	Hair follicle development/morphogenesis, epidermis and ectoderm development.
PN_SEA	FOXQ1	6	2	rs951318	A/G	0.919	Hair follicle development/morphogenesis, epidermis and ectoderm development.
SEA_IND	BX647987	4	1	rs1230209	T/G	0.71	/
SEA_MN	RGMB	5	2	rs1979980	A/C	0.48	Cell adhesion, transmembrane receptor protein serine/threonine kinase signaling pathway, BMP signaling pathway, regulation of transcription and nitrogen compound metabolic process.

Note: Groups are abbreviated as: JK – Japanese&Korean, Han – Han Chinese, SCT1 – SouthernChinese&Thai1, SCT2 – SouthernChinese&Thai2, INDO – Indonesian, PN – PhilippineNegrito, SEA – SoutheastAsian, MN – MalaysianNegrito, IND – Indian. Slash represents unknown gene function. After ranking candidates by averaging three SNPs that displayed higher F_ST_ within candidate genes, we put forward the top selection signal in each comparison of the nine groups, underlying the greatest possibility of population differentiation. No. SNP represents the number of SNPs within the gene. The function column shows the biological processes and phenotypic associations.

Specific to Philippine Negrito population, the top candidate gene *FOXQ1* showing the strongest signal of positive selection in comparisons with most other Asian groups was involved in the development and morphogenesis of the hair follicle. In Malaysian Negrito population, the outstanding candidate *WNT4* had something to do with gamete generation and specificity, indicating the influence of selection on human reproduction. Additional candidate genes specific to some groups were also identified (Table S6).

#### Outstanding signatures of local adaptation in Asia

**Figure 2:**
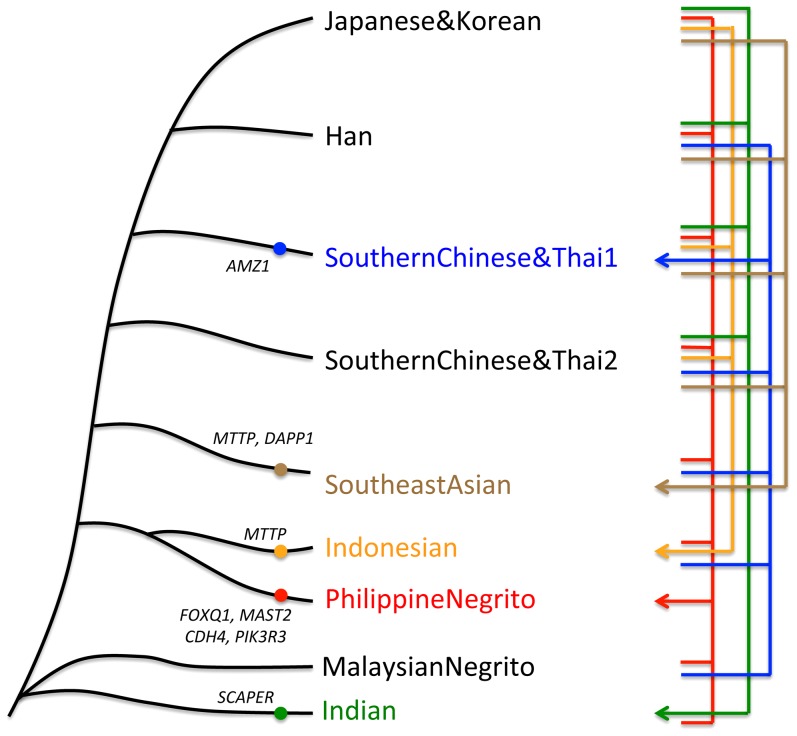
Outstanding candidate genes underlying local adaptation show consistency in related population groups. The genetic distance tree was constructed based on the global F_ST_ of those 9 groups. The genes shown in circles on the tree were selection signals specific to the corresponding group (as the arrows point). They presented great allele frequency differentiations in the comparisons of local group and other groups joint by the line of the same color (on the right) as the arrow.

**Figure 3:**
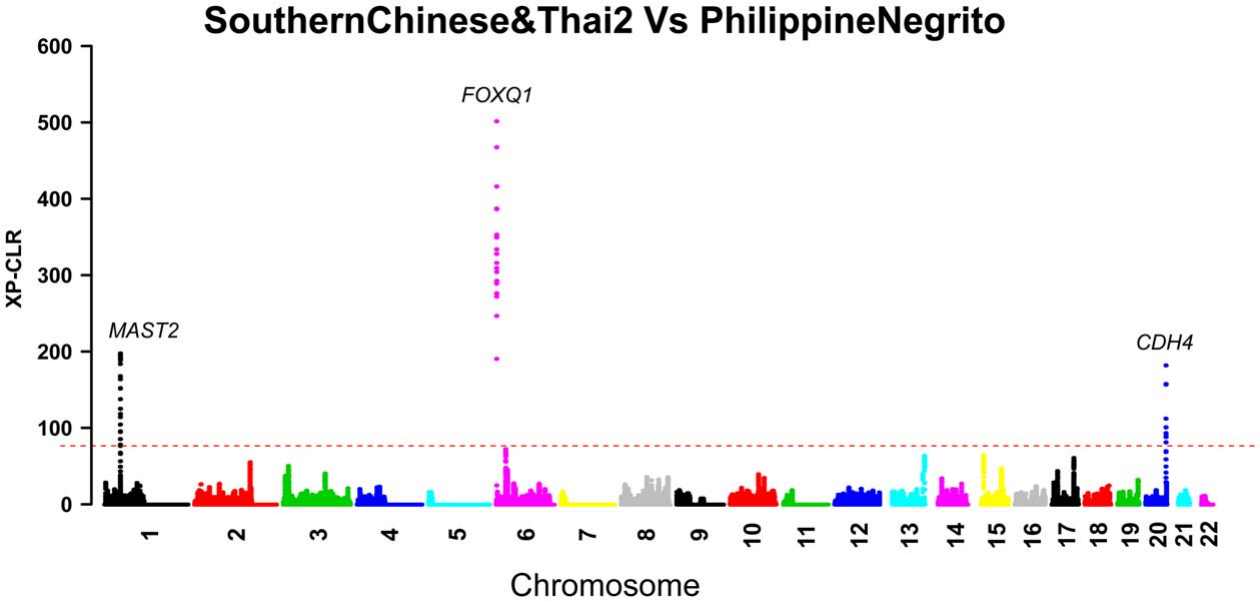
Signatures of *FOXQ1*, *MAST2*, and *CDH4* in the comparison of SouthernChinese&Thai2 and Philippine Negritos. XP-CLR score was calculated as depicted in the Methods. Against the whole-genome distribution of XP-CLR score, the strongest signals were *FOXQ1*, *CDH4* and *MAST2* in the comparison between Philippine Negritos and SouthernChinese&Thai2. The horizontal line indicates a top 50 genome-wide cutoff level.

Here, we worked out a list of candidate genes showing strong signatures of local adaptation in Asian populations (Table S7). We believe that these prioritized genes are more likely to be putative local adaptation signatures, since they are specific to a particular group or have occurred in closely related populations (Figure 2). Both in F_ST_ and XP-CLR analyses, we observed many strong signatures of group differentiation between Philippine Negritos and other Asian populations, among which the strongest one came from *FOXQ1* located on chromosome 6 encoding forkhead box Q1 protein which plays a role in hair follicle development and regulates epithelial-mesenchymal transition in human cancers [42], [43]. Other significant signals exhibiting Philippine Negrito specific trend encompassed *MAST2* on chromosome 1 and *CDH4* (cadherin4) on chromosome 20. The whole genomic picture of selective signals in these comparing pairs confirmed the most significant signature of local adaption in Philippine Negritos (Figure 3, Figure S1). Interestingly, we also observed the strong signal of *FOXQ1* in the comparisons between Philippine Negritos and all the other groups.

Apart from *FOXQ1*, we also identified a strong signature specific to Philippine Negritos using both F_ST_ and XP-CLR analysis. According to the F_ST_ test, within the *PIK3R3* gene on chromosome 1, which was one of the top ten candidate signals and just second to *FOXQ1* in every pair, one single SNP *rs10489769* showed strong statistical signature. In XP-CLR test, it was also the same SNP *rs10489769* that made *PIK3R3* one of the top 50 genes. Despite the fact that only the data of a single SNP was available to support the signal, the allele frequency of the SNP displayed significant divergence between Philippine Negritos and all the other Asian populations. *PIK3R3* regulates protein-tyrosine kinase activity and plays a crucial role in biological processes such as insulin stimulation, platelet activation, T cell costimulation, and blood coagulation [44]. Considering its extremely high scores under both F_ST_ and XP-CLR analysis, and its effect on immune protection and signal transduction under selective pressures, we suggested that *PIK3R3* would be an extremely strong signal and deserved to be further studied.

**Figure 4:**
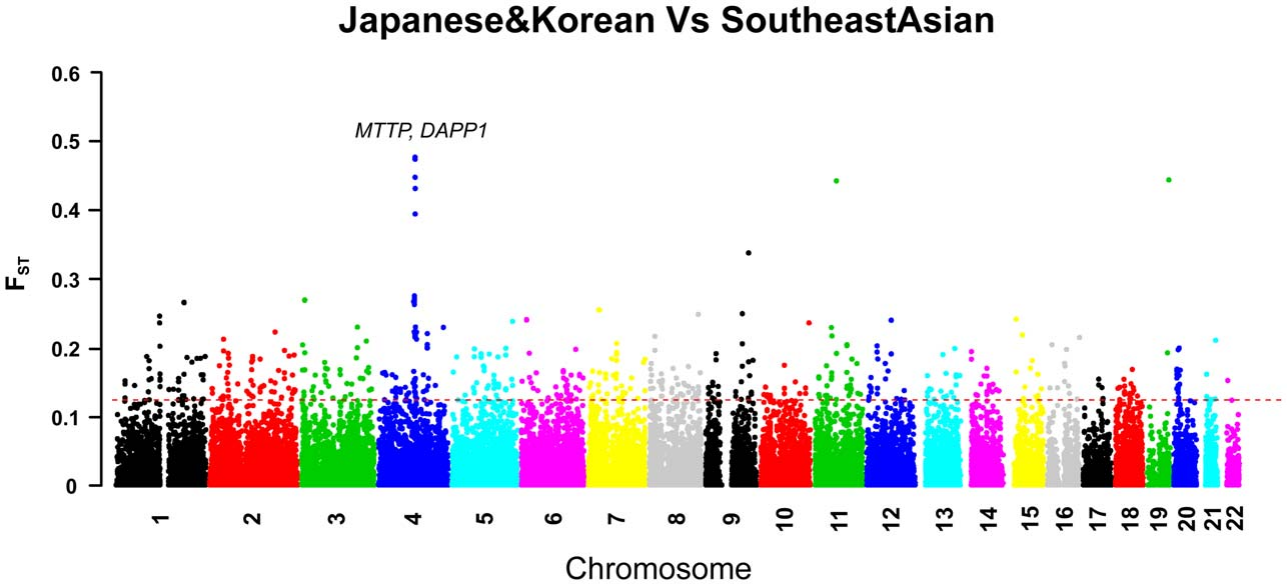
Signatures of *MTTP* and *DAPP1* in the comparison of Southeast Asian populations with Japanese and Koreans. SNP-specific F_ST_ statistic between Japanese&Korean populations and Southeast Asian populations was calculated for each genotyped SNP. *MTTP* and *DAPP1* on chromosome 4 showed significantly high F_ST_ values. The horizontal line indicates a 1% genome-wide cutoff level.

Additionally, a significant region on chromosome 4 containing *MTTP* and *DAPP1* exemplified the divergence between northern populations (Japanese&Korean, SouthernChinese&Thai1, SouthernChinese&Thai2, Han Chinese) and southern populations (Southeast Asian, Indonesian). *MTTP* protein catalyzes the transport of lipoproteins in the process of lipid metabolism and its variants are associated with plasma cholesterol levels and body mass index [45]. *DAPP1* protein, an adaptor protein, regulates antigen receptor signaling downstream of phosphatidylinositol 3-kinase and is closely related to human immune system [46]. The plots of their F_ST_ scores upon the whole genome loci in the comparisons between Southeast Asian populations and northern populations (Japanese&Korean, Han Chinese, SouthernChinese&Thai1, SouthernChinese&Thai2) showed remarkable evidence of selection signatures (Figure 4, Figure S2).

Re-considering those strong signals identified by F_ST_ analysis, we examined in pairs the list of top candidate genes against that of signal candidates supported by XP-CLR analysis (Table S2). Besides those candidates mentioned previously, we detected another strongest signature, *AMZ1*, which was also confirmed as one of the top 50 signals under XP-CLR analysis (Table S7). This protein was found to be a novel member of a family of metalloproteases with a zinc-binding site [47]. Moreover, although *SCAPER*, encoding S phase cyclin A-associated protein in the ER, did not show the strongest signal, it did have consistent results under both analyses in the comparison between northern populations (Han Chinese, SouthernChinese&Thai1, SouthernChinese&Thai2) and southern populations (Indian) (Table S7). This indicates much more space to explore functional implication and the mechanism of natural selection, especially the local adaptation of populations residing in different latitudes of Asia.

## Discussion

In this study, we performed the first comprehensive genome-wide scan for natural selection in 63 Asian populations and identified a number of putative selection signals. These may further the understanding of human local adaptation in Asia. One of the great advantages of our data is the fact that it was produced by sampling populations of a high geographic resolution and covering a large number of Asian populations, particularly Southeast Asian populations.

To draw a general picture of genome-wide signs of local adaptation in Asian populations, we investigated significant SNPs underlying population differentiation with respect to allele frequency. Using the sliding-window-based approach, we adopted a 1% genome-wide cutoff with respect to population differentiation across windows and indicated top 200 genes as putative selection signals in each group pair. Our results showed that Negritos and Indians had the most significant divergence regarding the allele frequency. Further, functional analysis of candidate genes suggested that natural selection among Asian populations is likely to induce mutations that play roles in alternative splicing. Selection divergence from northern Asian populations and southern Asian populations aimed at genes related to cadherin and a plenty of immune and related disease pathways. Another intriguing enrichment was about biological pathways of human height and growth, making Japanese and Korean populations different from Philippine Negritos. Moreover, Philippine Negritos and Malaysian Negritos demonstrated great genetic divergence and carried genes with polymorphisms whose allele frequency differed from other Asian populations.

Despite the fact that we did have observed some enrichment of genes, a part of categories, mainly for cell adhesion-related terms, do harbor many genes from protocadherin gamma gene cluster that are physically closely located in a region of 100 kb (Table S4). Although these genes might be under selective sweep due to linkage disequilibrium, it was also possible that they were independently affected by natural selection since these gene products, such as different protein isoforms, could physically interact with each other or form into protein complexes through biological networks. After all, a sort of enriched categories contained many genes not closely linked with each other on chromosomes, such as alternative splicing and immune associated pathways, indicating that the observed enrichment might not result from the physical linkage of chromosome position, but more likely from independent selection for genes involved in similar biological function and cellular pathways.

To confirm the reliability of the candidate signals identified by F_ST_ approach and better understand the selective signals, we turned to the results of XP-CLR analysis, and highlighted notable examples of candidate genes showing large numbers of extreme SNPs achieving peak F_ST_ and XP-CLR scores (Table S3). Generally speaking, XP-CLR test is much more powerful than F_ST_ approach in detecting signals with genome-wide data while it may also have some limits considering the low density of SNP markers, etc.. A number of genes showed region-specific signs of local adaptation in both approaches. The most striking example was *FOXQ1*, which was significantly different between Philippine Negritos and all the other Asian populations, and was accompanied by *PIK3R3* in the ranking list. Another remarkably strong signals, *MTTP* and *DAPP1*, displayed group differentiation between northern population (Japanese&Korean, SouthernChinese&Thai1, SouthernChinese&Thai2, Han Chinese) and southern populations (Southeast Asian, Indonesian), which was supported by F_ST_-based analysis. We believe that these genes merit further study because they are all important for human development or immunity, especially *PIK3R3*, since it is involved in the insulin receptor signaling pathway, which has been suggested as an adaptive pathway among African Pygmies [48].

In this study, our results provided a great deal of evidence for local adaptation in Southeast Asian populations that burden historical and geographical admixture, and in Negritos that have received increasing amounts of attention from the research community. Previous studies have identified some significant and comprehensive local adaptation signatures in African pygmies that are similar to Negritos on stature but differ substantially in genetic makeup. Most of the signatures lie in the immunity-related genes because of the different microbial environments in different habitats. For example, the *HLA* region varies dramatically across broad geographical populations, and it has been found to be a predictor of northern versus southern ancestry in Europe and other parts of the world [49]. In the present study, *HLA* showed notably high F_ST_ and XP-CLR scores in the comparisons of certain Asian populations, especially between Indians and SouthernChinese&Thai1, and between Han Chinese populations and southeastern populations (Indonesian, SouthernChinese&Thai2) (Table S3, Table S5). Our functional analysis showed that the immunoglobulin domain is enriched in candidate signals that differ among Asian populations. Additionally, stature is a highly visible trait that was likely to be subjected to natural selection. Many genes have been found related to height variance in African pygmies [48], some of which were also identified in our study (Table S8). Especially, *IFNG* and *LEPR* reflected the difference between northern and southern Asian populations. As people who reside at higher latitudes (north) are generally taller than those living at lower latitudes (south), these two genes may be in association with the stature of Asian individuals.

Considering the differences between northern and southern populations, the most remarkable and comprehensive clue may be related to skin pigmentation. It has been proved, from Africa to eastern Europe and eastern Asia, that microRNA regulation acts as a rheostat to optimize *TYRP1* expression in response to differential UV radiation based on latitude [50]. Also, *DDB1*, which protects the skin from solar UV exposure, has different alleles fixed in continents from different latitudes [51]. But no pigmentation-related gene was identified in our study.

However, this study has two principal limitations. The first is the low SNP density relative to the high-density data available in public resources and therefore may not fully represent the entire genome. But these SNP markers randomly distribute across the genome, thus providing a background of genome-wide information [39]. So the population genomics approach we adopted in this study could distinguish between population demographic history and natural selection, allowing us to identify ‘outliers’ as candidates underlying selection [3]. Furthermore, with respect to linkage disequilibrium, the SNP markers could be considered as tag SNPs for genes underlying natural selection. Because of this, a more accurate sign of local adaptation might be located at a region adjacent to the significant signals we suggested. The second is the difficulty of presenting our results because the data cover a wide range of populations and groups. We tried to summarize our findings in a reasonable and clear manner but unavoidably missed some details.

Despite its limitations, our study can be regarded overall as a useful guide for further evaluation of local adaptation in Asian populations. The strongest signs of local adaptation in Asian populations may have been novel candidates, suggesting the advantage of data, such as the data evaluated here, which cover wide geographic areas within Asia. This may be particularly true of candidates discovered among the Negritos. A more comprehensive collection of greater numbers of samples across a wider geographic range will be necessary for further studies of positive selection, especially in the populations whose members exist across special geographic regions, such as the Negritos and Tibetans. More importantly, high-resolution data such as genome-wide SNP data and next generation sequencing data will facilitate investigation of the genetic basis of local adaptation in a more effective and detailed way than has previously been possible. The development of novel and sophisticated methods of deciphering signs of natural selection will assist researchers and improve the biological understanding of human adaptive evolution.

## Methods

### Data collection

The HUGO Pan-Asian SNP consortium constructed a database (PanSNPdb) containing data of human genetic diversity among Asians by sampling 1,719 unrelated individuals among 71 populations from China, India, Indonesia, Japan, Malaysia, the Philippines, Singapore, South Korea, Taiwan, and Thailand [38]. We collected 54,794 autosomal single-nucleotide polymorphisms (SNPs) in 1513 individuals genotyped by Affymetrix GeneChip Human Mapping 50K Xba Array from this database.

### F_ST_ analysis

To detect signs of local adaptation in Asian populations, we used an allele-frequency-based approach. For each pair of groups, we calculated unbiased estimates of F_ST_ following Weir and Hill [52] based on 54,794 SNP markers. We set sliding windows with the size of 500 kb along the genome, and those containing less than 5 genotyped SNPs (about 20% of all windows) were filtered out. Then we calculated the average F_ST_ value of the top 3 SNPs in each window as the value of it and defined 1% tail of the distribution as the threshold. Those windows above were considered regions underlying local adaptation. After that, for each individual window, we sorted SNPs based on their F_ST_ and selected the top n SNPs (n = int(N/i), N represents the total number of SNPs in a certain window. If N<20, i = 5; if N> = 20, i = 10), which were thought to be candidate SNPs. Finally we mapped these candidate SNPs onto genes and picked out the top 200 genes as candidate genes for local adaptation.

### XP-CLR testing

To confirm selection signals identified by F_ST_ analysis, we applied another allele-frequency-based method: the cross-population composite likelihood ratio test (XP-CLR) [40]. It is not affected by ascertainment bias and has the advantage of enlarging signals. We selected 50 top signal windows containing on average tens of SNPs in each pair as the putative signals underlying local adaptation.

### Functional Annotation

We implemented DAVID Bioinformatics Database (http://david.abcc.ncifcrf.gov/) to perform functional analysis upon candidate genes acquiring significant SNPs in each pair of group comparisons [53], [54]. We assessed their functional enrichment in terms of OMIM disease, Gene Ontology, SP_PIR_KEYWORDS, INTERPRO, PANTHER pathway and KEGG pathway (Table S4). Here, we used false discovery rate (FDR) to correct multiple tests developed by Benjamini [55].

## Supporting Information

Figure S1 **Signature of local adaptation in Philippine Negritos adjacent to** ***FOXQ1*** **.**
(PDF)

Figure S2 **Signatures of local adaptation in Southeast Asian populations associated with** ***MTTP*** **and** ***DAPP1*** **.**
(PDF)

Table S1 **Characterization of datasets and group division.**
(PDF)

Table S2 **Selection candidates detected by XP-CLR approach.**
(XLSX)

Table S3 **Candidate genes overlapping with XP-CLR genes.**
(XLS)

Table S4 **Functional enrichment analysis.**
(XLSX)

Table S5 **Top 10 candidate genes in 36 comparing pairs.**
(XLS)

Table S6 **Candidate genes underlying local adaptation identified by F_ST_ approach.**
(XLS)

Table S7 **Outstanding candidate genes demonstrating strong signs of local adaptation in Asia.**
(XLS)

Table S8 **Candidate genes overlapping with 14 literature genes.**
(XLS)

Text S1 **Results of XP-CLR analysis.**
(PDF)
