# Supplementary material for: Genome-Wide Landscapes of Human Local Adaptation in Asia
Source: PLoS One. 2013 Jan 22;8(1):e54224. doi: 10.1371/journal.pone.0054224 (PMC3551950; doi:10.1371/journal.pone.0054224)

Figure S1. Signature of local adaptation in Philippine Negritos adjacent to *FOXQ1*

A. XP-CLR analysis

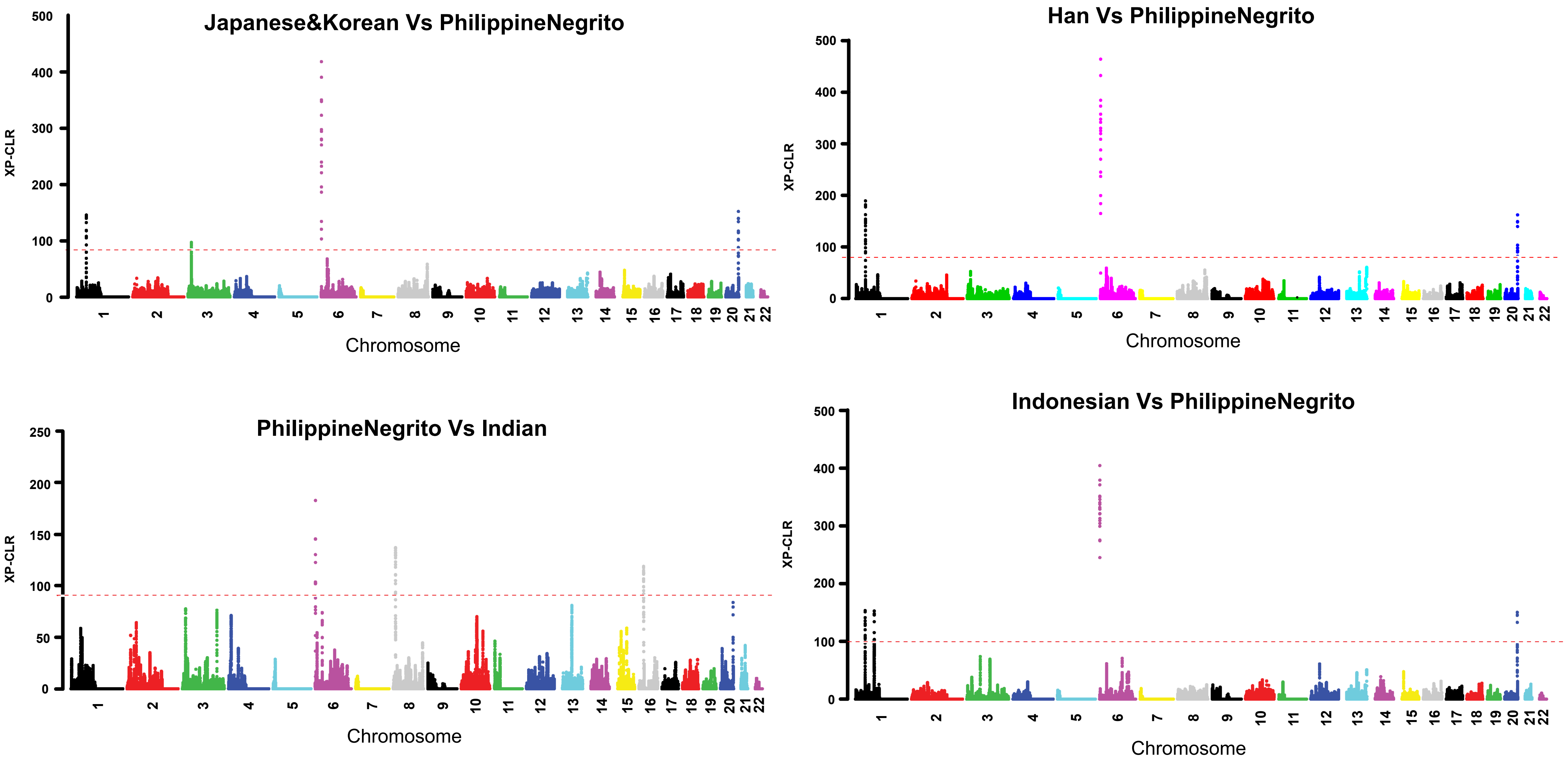

B. Window-based F_{ST} analysis

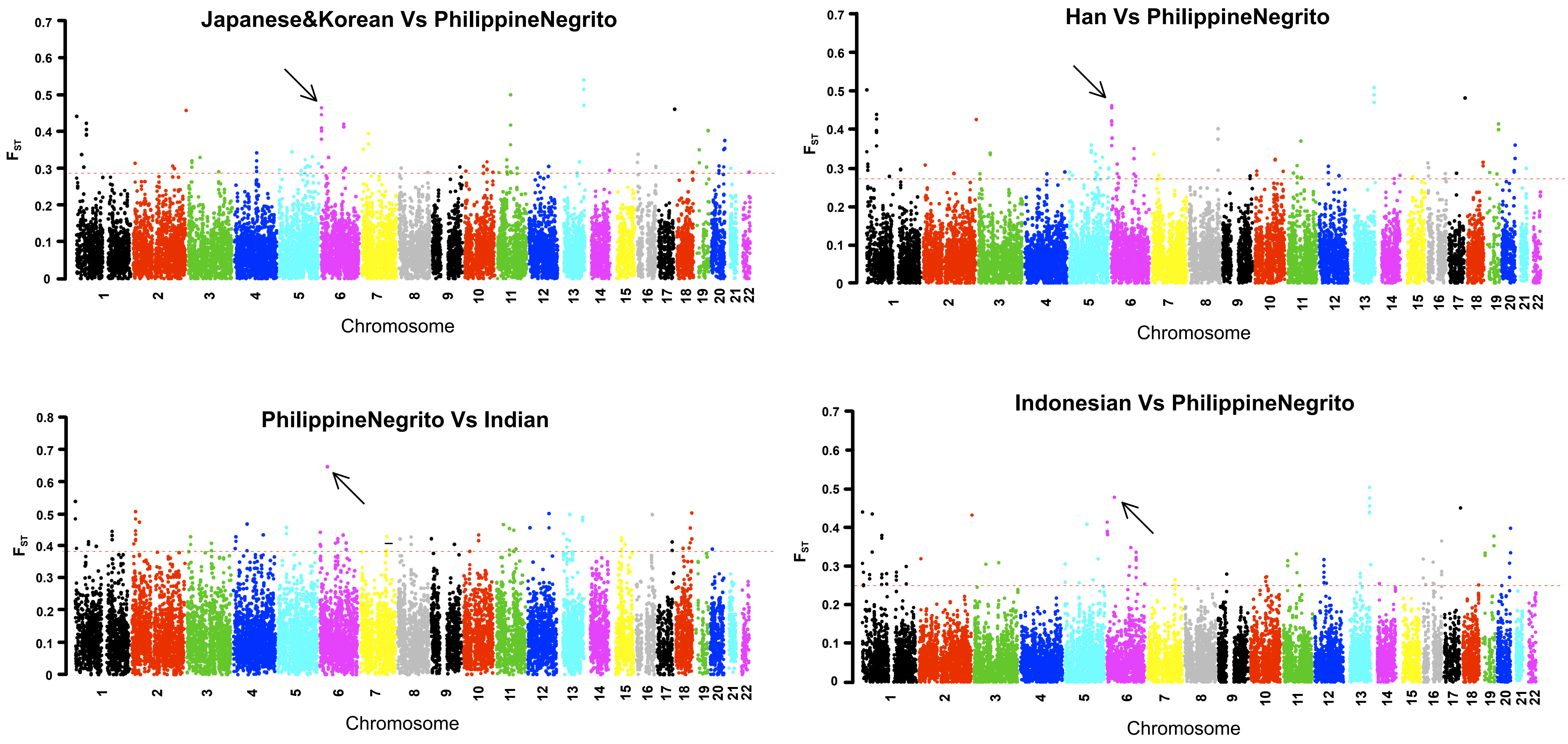

Supplement: Figure S1 — Signature of local adaptation in Philippine Negritos adjacent to FOXQ1 . (PDF) [file pone.0054224.s001.pdf]
