# Supplementary material for: Genome-Wide Landscapes of Human Local Adaptation in Asia
Source: PLoS One. 2013 Jan 22;8(1):e54224. doi: 10.1371/journal.pone.0054224 (PMC3551950; doi:10.1371/journal.pone.0054224)

Figure S2. Signatures of local adaptation in Southeast Asian populations associated with *MTTP* and *DAPP1*

A. Window-based F_{ST} analysis

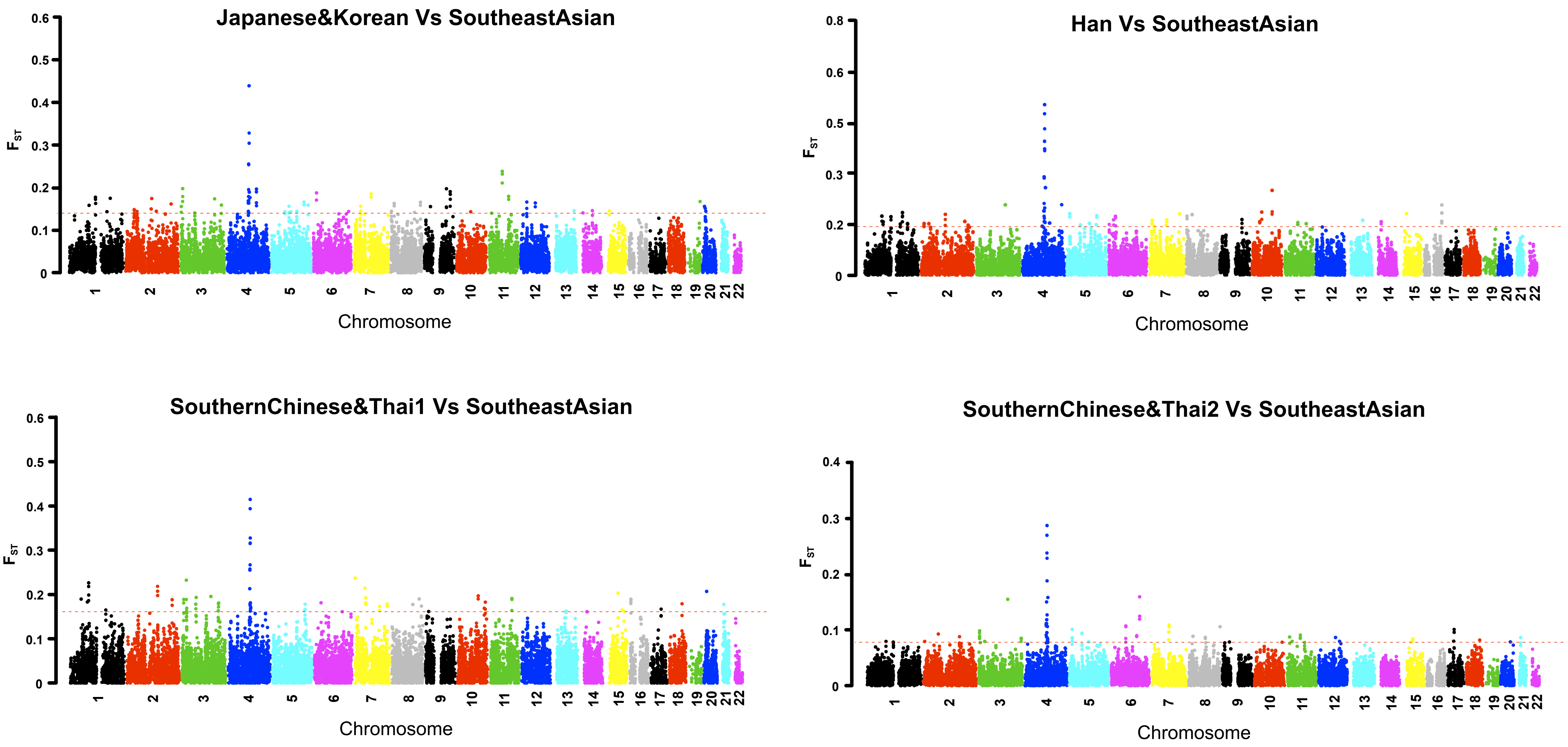

B. SNP-based F_{ST} analysis

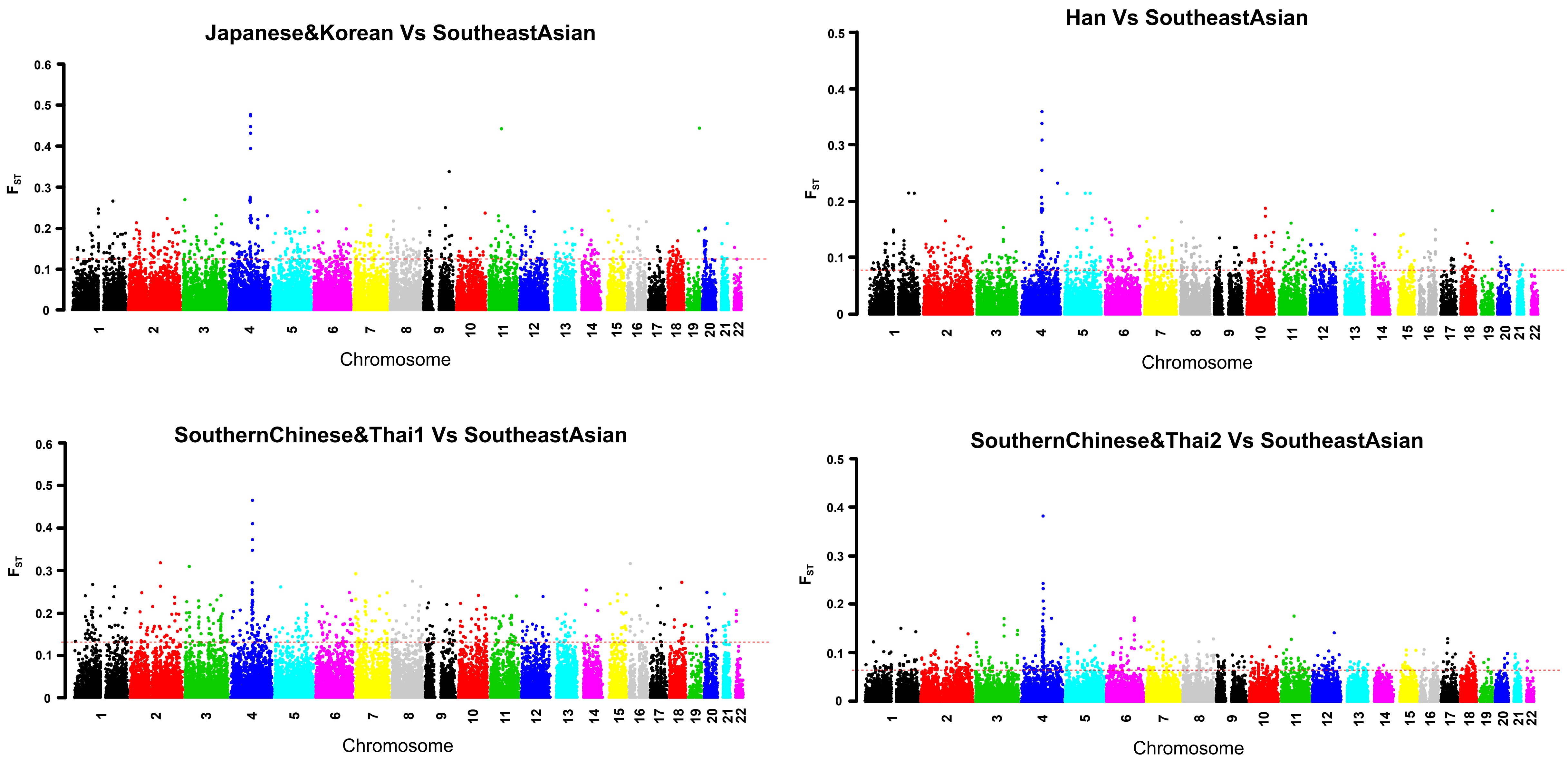

Supplement: Figure S2 — Signatures of local adaptation in Southeast Asian populations associated with MTTP and DAPP1 . (PDF) [file pone.0054224.s002.pdf]
