# Supplementary material for: Genome-Wide Landscapes of Human Local Adaptation in Asia
Source: PLoS One. 2013 Jan 22;8(1):e54224. doi: 10.1371/journal.pone.0054224 (PMC3551950; doi:10.1371/journal.pone.0054224)
Supplement: Table S1 — Characterization of datasets and group division. (PDF) [file pone.0054224.s003.pdf]

Table S1 Characterization of datasets and group division

Group Name	ID	Location	Latitude	Longitude	Ethnicity	size	total
Japanese&Korean	JP-RK	Japan	26.5	127.9	Ryukyuan	49	254
	JP-ML	Japan	35.7	139.8	Japanese	71	
	JPT	Japan	35.7	139.8	Japanese	44	
	KR-KR	Korea	36.9	127.5	Korean	90	
Han	CHB	China	40	116.4	Han	45	206
	CN-SH	China	31.2	121.5	Han	21	
	TW-HA	Taiwan	25	121.5	Han	48	
	TW-HB	Taiwan	25	121.5	Han	32	
	SG-CH	Singapore	1.4	103.8	Han	30	
	CN-GA	China	23.3	113.5	Han	30	
SouthernChinese &Thai1	CN-HM	China	26.3	108.7	Hmong	26	65
	TH-HM	Thailand	18.6	98.1	Hmong	20	
	TH-YA	Thailand	20	100.2	Yao	19	
SouthernChinese &Thai2	CN-CC	China	24.4	110.2	Zhuang	26	329
	CN-JI	China	18.9	109.8	Jiamao	31	
	TH-TL	Thailand	19.2	100.9	Tai Lue	20	
	TH-TY	Thailand	18.4	98.9	Tai Yong	18	
	TH-TK	Thailand	18.6	98.9	Tai Kern	18	
	TH-TU	Thailand	19	99	Tai Yuan	20	
	TH-MA	Thailand	18.7	100.5	Mlabri	18	
	TH-TN	Thailand	19.1	100.9	H'Tin	18	
	TH-PP	Thailand	20.4	99.9	Plang	18	
	CN-WA	China	22.8	100.2	Wa	56	
	TH-LW	Thailand	18.4	98.1	Lawa	19	
	TH-KA	Thailand	18	98.4	Karen	20	
	CN-JN	China	22	101	Jinuo	29	
	TH-PL	Thailand	19.9	99.2	Palong	18	
Indonesian	AX-ME	Pacific	-5.8	155.1	Melanesian	5	119
	ID-AL	Indonesia	-8.3	124.7	Alorese	19	
	ID-LE	Indonesia	-8.3	124.7	Lembata	19	
	ID-LA	Indonesia	-8.3	123	Lamaholot	20	
	ID-SO	Indonesia	-8.6	120.1	Manggarai	19	
	ID-RA	Indonesia	-8.7	120.5	Manggarai	17	
	ID-SB	Indonesia	-9.8	120	Kambera	20	
PhilippineNegrito	PI-AG	Phillippines	13.7	123.3	Negrito	8	67
	PI-AE	Phillippines	14.9	120.2	Negrito	8	
	PI-MW	Phillippines	9.7	125.6	Negrito	19	
	PI-IR	Phillippines	13	121.1	Negrito	9	
	PI-AT	Phillippines	11.9	122	Negrito	23	
SoutheastAsian	AX-AM	Taiwan	23.7	121.4	Ami	10	261
	AX-AT	Taiwan	24.6	121.4	Atayal	10	
	PI-UB	Phillippines	17.2	121.9	Urban	20	
	PI-UN	Phillippines	14.6	121	Urban	19	
	PI-UI	Phillippines	6.9	122.1	Urban	20	
	PI-MA	Phillippines	8.2	125.9	Manobo	18	
	ID-MT	Indonesia	-0.3	98.4	Mentawai	15	
	ID-TR	Indonesia	-4.7	119.7	Toraja	20	
	ID-ML	Indonesia	-3	104.7	Malay	12	
	ID-KR	Indonesia	1.5	100	Batak Karo	17	
	ID-TB	Indonesia	2.3	99.1	Batak	20	
	ID-DY	Indonesia	1.2	116.7	Dayak	12	
	MY-MN	Malaysia	2.8	102.2	Malay	20	
	SG-MY	Singapore	1.4	103.8	Malay	30	
	MY-KN	Malaysia	5.3	102	Malay	18	
MalaysianNegrito	MY-JH	Malaysia	5.4	101.1	Negrito	50	80
	MY-KS	Malaysia	5.7	100.9	Negrito	30	
Indian	IN-DR	India	15.3	77.8	Upper-caste	24	132
	IN-WI	India	26.7	74	Bhil	25	
	IN-EL	India	23	88.2	Upper-caste	16	
	IN-SP	India	29.1	76.5	Upper-caste	23	
	IN-WL	India	19.7	75.9	Upper-caste	14	
	IN-NL	India	26.8	81.4	Upper-caste	15	
	IN-IL	India	26.7	74	Upper-caste	15	
