# Supplementary material for: Genome-Wide Landscapes of Human Local Adaptation in Asia
Source: PLoS One. 2013 Jan 22;8(1):e54224. doi: 10.1371/journal.pone.0054224 (PMC3551950; doi:10.1371/journal.pone.0054224)
Supplement: Text S1 — Results of XP-CLR analysis. (PDF) [file pone.0054224.s011.pdf]

# The Results of XP-CLR analysis

## Overview of Signals

To confirm those loci underlying selection identified by $F_{ST}$ analysis, we conducted another allele-frequency-based method called cross-population composite likelihood ratio test (XP-CLR) by comparing those nine groups in pairs [47]. First, we calculated population differentiation statistic XP-CLR score upon genome-wide markers for each group pair. Here, we selected 50 top signal windows containing less than 100 SNPs on average in each pair, respectively, as the putative signals underlying local adaptation. To delineate the overall picture of putative signals of local adaptation in the 9 groups, we calculated average XP-CLR score and maximum XP-CLR score of signals underlying selection and located candidate genes whose SNP markers were selected as putative signals in each group pair comparison (Table S2).

Not surprisingly, the northern part of Asian displayed remarkable population differentiation compared with the southern part. Interestingly, the results from Table S2 showed that Asian Negritos from both the Philippines and Malaysia have much more significant genetic differences than with other Asian populations.

## Functional Analysis

In order to further characterize local adaptation in 63 Asian populations, we conducted functional enrichment analysis towards those candidate genes whose SNP markers exhibited extremely significant population differentiation over the genome.

On one hand, we tried to understand the general characteristics of local adaptation in most Asian populations. Functional annotation (see Method) revealed that the functional category of immunity was most frequently presented in compared group pairs, which is consistent with some previous findings and followed by the transmembrane region and the connection peptide, suggesting that natural selection played a vital role in those aspects upon most Asian populations, leading to local adaptation. These three categories were most significantly enriched in the group pairs

involving East Asian populations (e.g. Japanese, Korean and Han Chinese), South Asian populations (SouthernChinese&Thai1, SouthernChinese&Thai2 and Indian) and Southeast Asian populations (Indonesian and Philippine Negritos).

On the other hand, group specific or regional selection signals were very impressing. The comparison between Indonesian and SouthernChinese&Thai1, SouthernChinese&Thai2, and Han, respectively, revealed that *HLA* could have significant difference between them, meaning that the immune system might have been affected by natural selection. Also, the divergence of Negrito populations of the Philippine from most of the other Asian populations, which showed that *MAST2* exhibited an extremely strong signal, could be another evidence for this conclusion. Besides, when comparing Malaysian Negritos with some other populations (Han and Japanese&Korean), we found that the *OR* gene family exhibited relatively strong signatures, from which it can be inferred that Malaysian Negritos share few similarity with others on the structure or function of some transmembrane regions, since *OR*s belong to G-protein-coupled receptor (GPCR) with 7 alpha-helix transmembrane proteins in it.

In summary, giving clues to biological basis of regional adaptation, our functional analysis provided much more evidences on variations of biological function between northern and southern populations in Asia, as well as such specific populations as Philippine Negritos and Malaysian Negritos. The details are given in the following section.

## Identification of candidate genes underlying local adaptation

As many previous studies revealed, the immune system might have suffered natural selection during its evolution because of the ecological divergence and the change of human lifestyle, and here is another proof for it. The *HLA*s (human leukocyte antigen), including *HLA-A*, *HLA-G*, *HLA-G2.2*, *HLA-G3* and *HLA-H*, on chromosome 6 showed a great difference between Indonesian and some other Asian populations (SouthernChinese&Thai1, SouthernChinese&Thai2 and Han). *HLA*s encode cell-surface antigen-presenting proteins and the their diversities in the human

population is one aspect of disease defense. Besides, the XP-CLR score of *HLA*s between Indonesian and Han (maximum XP-CLR score: 70.078, average XP-CLR score: 50.971) is much higher than that between Indonesian and SouthernChinese&Thai1 (maximum XP-CLR score: 40.015, average XP-CLR score: 37.533) or SouthernChinese&Thai2 (maximum XP-CLR score: 43.509, average XP-CLR score: 41.353), reflecting the divergence between the northern and southern part of Asian.

Meanwhile, we observed many strong signatures of group differentiation between Philipppine Negritos and the other Asian populations. The strongest one underlying selection came from *FOXQ1* located on chromosome 6 encoding forkhead box Q1 protein which plays a role hair follicle development and regulates epithelial-mesenchymal transition in human cancers [49,50]. The XP-CLR score of *FOXQ1* reached top in the group pair of Philippine Negritos and SouthernChinese&Thai1 (maximum XP-CLR score: 495.806, average XP-CLR score: 330.696). Other significant signals exhibiting Philippine Negrito specific trend encompassed *MAST2* on chromosome 1, encoding microtubule-associated serine/threonine-protein kinase 2 which interacts with other proteins in signal transduction [51], and *CDH4* (Cadherin4) on chromosome 20 which is thought to be a candidate tumor repression gene on gastroenteric tumor and nasopharyngeal carcinoma [52], suggesting that the mechanism of tumorigenesis might have experienced a differentiation during its evolution. Additionally, the top XP-CLR score of *MAST2* (maximum XP-CLR score: 195.550, average XP-CLR score: 140.601) and *CDH4* (maximum XP-CLR score: 180.050, average XP-CLR score: 121.508) were both in the group pair of Philippine Negritos and SouthernChinese&Thai2. The whole genomic picture of selective signals in these comparing pairs confirmed the most significant signature of local adaption in Philippine Negritos (Figure S1).

When comparing populations from Malaysian Negrito group with those from Han and Japanese&Korean, respectively, we found that, on chromosome 11, several genes of olfactory receptor gene superfamily, such as *OR5AR1*, *OR5M10*, *OR5M11*, *OR5M9*, *OR5R1*, *OR8J1*, *OR8U8* and *OR9G9*, continuously obtained higher rank

scores with the maximum XP-CLR score of 64.971 between Japanese&Korean and Malaysian Negrito. Meanwhile, we did not see this signal in the comparisons among the other groups. Olfactory receptors interact with odorant molecules in the nose to initiate a neuronal response that triggers the perception of a smell, suggesting that Han Chinese, Japanese and Korean share more similarity than with Malaysian Negrito on olfactory. Therefore, we argue that *OR* gene superfamily must play an important role in evolution, causing local adaptation upon populations of Malaysian Negritos.
